# Does trajectory matter? A study looking into the relationship of trajectory with target engagement and error accommodation in subthalamic nucleus deep brain stimulation

**DOI:** 10.1007/s00701-017-3151-y

**Published:** 2017-03-30

**Authors:** David Anthony Steel, Surajit Basu

**Affiliations:** 1 University of Nottingham Medical School, Nottingham, UK; 2 Department of Neurosurgery, Nottingham University Hospitals, Nottingham, UK

**Keywords:** Deep brain stimulation, Parkinson’s disease, Subthalamic nucleus, Targeting, Trajectory

## Abstract

**Background:**

Deep brain stimulation (DBS) of the subthalamic nucleus (STN) is now a key treatment choice for advanced Parkinson’s disease. The optimum target area within the STN is well established. However, no emphasis on the impact of trajectory exists. The ellipsoid shape of the STN and the off-centre traditional target point mean that variation in the electrode inclination should affect STN engagement. Understanding of this relationship could inform trajectory selection during planning by improving STN engagements and margins for error.

**Method:**

We simulated electrode placement at the clinical target through a set of trial trajectories. Twelve three-dimensionally reconstructed STNs were created from magnetic resonance imaging data of six patients. An appropriate target within each STN was then chosen. Each STN was approached through 56 simulated trajectories arranged in a grid covering a quadrant of skull around and in front of the coronal suture. A subset of 20 viable trajectories was reassessed for depth of engagement in each STN whilst approaching the chosen target.

**Results:**

Group averages for each trajectory are presented as traffic light maps and as an overlaid skull mask illustrating recommended electrode entry sites. Trajectories under 30 degrees anterior to the bregma and between 10 to 30 degrees off the midline accommodated over 2.4 degrees of wobble. A mean engagement of 6 mm was possible in half of the subset. The longest engagements are on trajectories which saddle the coronal suture, extending to 40 degrees lateral. Microelectrode tracts of 14 additional STNs were collated using the above protocol and engagement exceeded 5 mm in all central trajectories without capsular side effects, suggesting placement away from STN borders.

**Conclusions:**

Trajectory selection influences engagement and flexibility to accommodate electrode wobble or brain shift whilst approaching a chosen STN target. We recommend having the first trial trajectory 20 degrees anterior to the bregma, moving postero-laterally in successive trials to balance both error and engagement. When wider margins for error are beneficial (e.g. second side during bilateral procedures), trajectories nearer the coronal suture and around 25 degrees off the midline are advised.

## Introduction

Deep brain stimulation (DBS) of the subthalamic nucleus (STN) is an established method of managing symptoms of advanced Parkinson’s disease. The surgical procedure has evolved over the years from indirect targeting of the STN by ventriculography to present day direct target visualisation in high-resolution multimodality magnetic resonance imaging (MRI) studies, image fusion and computer assisted planning.

Whilst planning for STN-DBS, the surgeon selects the most appropriate target within the STN and aims to select a safe trajectory that avoids intersecting vital structures. Otherwise, surgeons differ in how they select their final approach. The authors posit that the non-spherical shape of the STN may allow for more tailored planning for two reasons:

‘Default’ trajectories suggested by planning software often transect important anatomical structures and therefore it is beneficial to gauge the ‘margin for error’ and engagement attributes of alternative entry points.

Patient outcomes improve with optimum electrode placement within the STN. The non-uniform shape of the STN suggests that the trajectory selected pre-operatively should directly influence engagement. This is compounded by the eccentricity of the traditional target point within the STN.

By the same reasoning, the likelihood of missing the STN should also vary with trajectory. Thus, giving trajectories different ‘margins for error’.

The importance of accurate targeting is well recognised. However, there is currently a lack of clarity on what should be the best trajectory to engage the STN during DBS surgery. Insight into how length of engagement and margin for error vary by trajectory could influence planning methodology with a view to improving patient outcomes. We sought to shed light on this relationship though analysis of serial virtual implantations.

## Materials and methods

### Patients

Data from the 12 STNs of six randomly selected patients who underwent DBS surgery were included in the study. Each patient had multimodality DBS imaging undertaken as part of routine clinical protocol (*see* Table 1 for imaging specifications). Appropriate permission was obtained to undertake this retrospective analysis of the clinical data.

**Table 1:** Imaging specifications

Modality	Details
CT	Slice thickness, 1.5 mm; 120kV/300 mA; cycle time, 2 s; rotation time, 0.75 s; standard resolution
MRI (T2)	Slice thickness, 2 mm; voxel size, 0.78 mm × 0.78 m; TSE factor, 15; TE, 80 ms; TR, 3,000 ms; NSA, 3
MRI (IR)	Slice thickness, 2 mm; voxel size, 0.72 mm A-P, 0.91 mm right-to-left; TSE factor, 7; TE, 15 ms; TR, 3,198 ms; NSA, 2; IR-delay, 400 ms
MRI (SWI)	Slice thickness, 0.5 mm; voxel size, 1 mm A-P, 0.99 mm right-to-left; TE, 22 ms; TR, 15 ms; NSA, 1
MRI (MP-RAGE)	Voxel size, 1 mm × 1 mm

*A-P* antero-posterior, *IR* inversion recovery, *MP-RAGE* magnetisation-prepared rapid gradient echo, *NSA* number of signal averages, *SWI* susceptibility weighted imaging, *TE* echo time, *TR* repetition time

, *TSE* turbo spin echo

### Virtual reconstruction

Imaging data were transferred to a surgical neuronavigation planning station and accessed via proprietary DBS planning software. Computed tomography (CT) and MRI (T1-weighted, T2-weighted and susceptibility weighted imaging [SWI]) scans were merged and each STN was virtually reconstructed using interactive segmentation. We used susceptibility-weighted MRI to delineate the STN boundaries, as this method was validated by our team previously by microelectrode recording studies [3]. The three-dimensional (3D) skull image was modelled by manual thresholding. Finally, the skull and STN models were set to one-tenth opacity, so that the probe could be fully visualised during simulated implantation.

### Target selection

We used the same targets within the STNs for this study as were used to place DBS electrodes in each patient’s procedure. The target coordinates for each patient were saved within the planning workstation and could be retrieved.

### Trajectory matrix

A trajectory matrix was formulated over the area of skull covering probable surgical entry points as well as more hypothetical trajectories for a more complete dataset. The battery of 56 trajectories, each spaced by 10 degrees in Cartesian axes, covered a quadrant of each skull just over and in front of the coronal suture. All trajectories converged towards the traditional STN-DBS target point. They were individually defined by the relationship between their path and a reference trajectory with a bregmatic entry point. Each trajectory was thus described using two angles, in the coronal and sagittal planes, corresponding to the arc and ring of the Cosman-Robert-Wells stereotactic frame, respectively.

### Simulated implantation

Simulated electrode implantations were carried out at each of the 56 trajectories on all 12 STNs. Within these trajectories two factors were assessed:

1. Margin for error in terms of accommodating for electrode wobble or brain shift
2. STN engagement in a subset of trajectories for which margin for error was reasonable

**Fig. 1:**
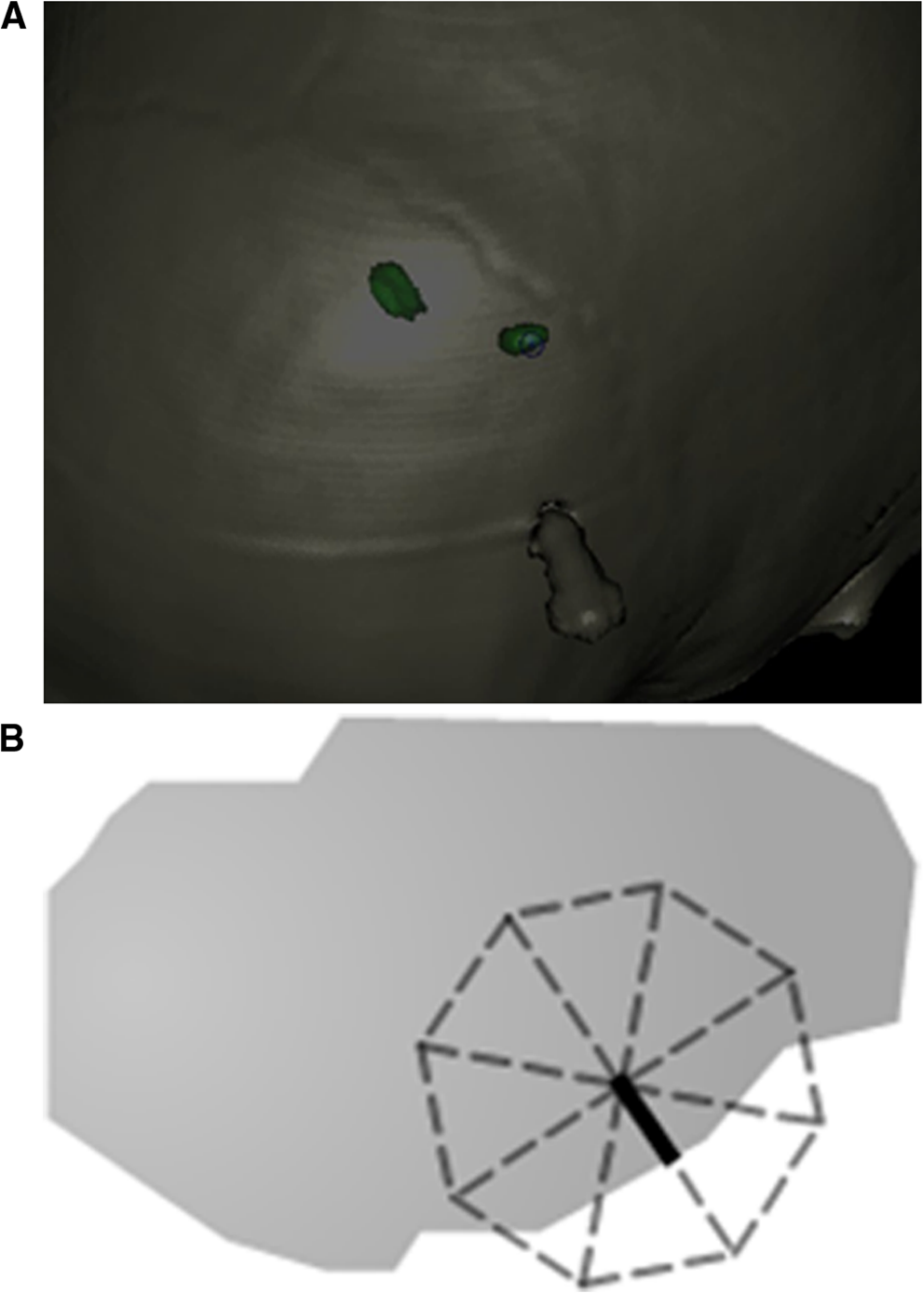
**a** ‘Probe’s eye view’ used for measurement of error margin. Screenshot from a StealthStation S7 Surgical Navigation System with Framelink software (Medtronic, Minneapolis, MN, USA). **b** In the blown-up schematic, the centre of the *dashed outline* represents the probe and the *solid line* is the distance recorded for this trajectory

Margin for error was measured by the distance from the electrode to the closest STN border in a probe’s eye view (Fig. 1). This measurement represents the radius of an imagined circle within which the electrode will engage the STN, which was then doubled and recorded as a diameter in millimetres. If the STN was missed by the electrode, the diameter was recorded as 0 mm. Entry point to target point distance and margin for error diameter were used to calculate an angular margin for error by trigonometry. Due to the nature of the software, error margin measurements were bound to the most superficial surface visible. That is, as our measurements were made with the skull overlay, the recorded distances were between two points in 3D space that took into account the curvature of the skull. If the skull overlay had been absent, then the less flat surface of the STN would have been most superficial, lending to more distorted measurements. Ideally an artificial flat plane overlying each STN would have been used for these measurements, but this was not possible in the simulator.

**Fig. 2:**
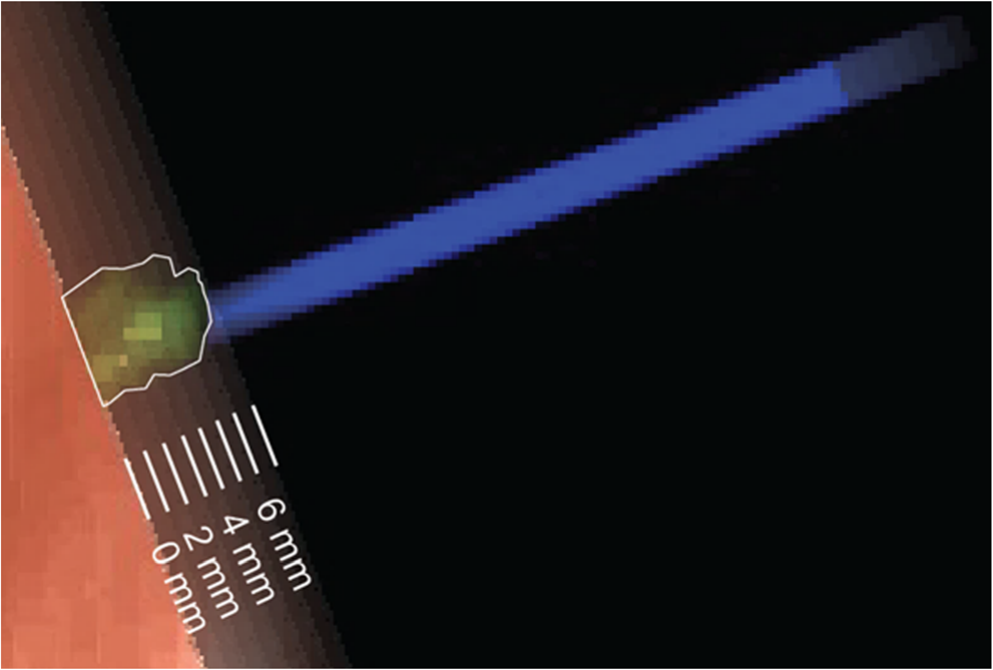
Obliquely cut MRI slices whose depths increase consecutively by 1 mm, transparently overlaid for illustration, such that the STN is eventually obscured and a minimum length of engagement can be determined. In this example, the recorded minimum engagement would be 6 mm. (Screenshot from a StealthStation S7 Surgical Navigation System)

The length of STN engagement was measured by reducing the depth of an opaque obliquely cut MRI slice from the target point until the probe’s intersection with the STN was barely visible (Fig. 2). This depth was then subtracted from the target point depth to give a minimum engagement value in millimetres.

## Results

### Margin for error

Margin for error was greatest, accepting a wobble of approximately 3.0 degrees, within an island of trajectories centred between 20 and 30 degrees laterally and 0 to 20 degrees anteriorly (Table 2). Sixteen trajectories had margins for error of 2.5 degrees or more with margin for error tailing off at more antero-lateral and postero-medial trajectories.

**Table 2:**
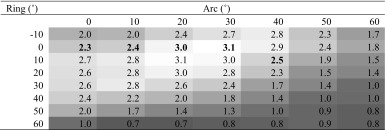
Mean angular margin for error in degrees for each electrode trajectory with greyscale formatting. Trajectories that approximately overlie the coronal suture are in *bold*

Trajectories that approximately overlie the coronal suture are in bold

### Mean engagement

In assessing mean engagement trajectories with poor margins for error, which tended to reside mostly in the periphery of the quadrant, were excluded. The most posterior row of trajectories and those overlying the superior sagittal sinus were also excluded. It was thought that the most posterior trajectories would not be feasible due to the underlying motor cortex. The resulting subset of 20 trajectories was investigated for their mean engagements with the STN (Table 3).

**Table 3:**
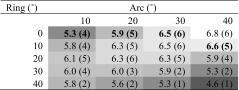
Mean engagement length in millimetres for each trajectory within the subset

*In brackets* are the minimum recorded engagement lengths in millimetres for each trajectory within the subset. Trajectories that approximately overlie the coronal suture are in *bold*

An antero-medial to postero-lateral strip of highly engaging trajectories was found, with the latter end having the most highly engaging trajectories. Mean engagement exceeded 5.0 mm in all but the single most antero-lateral trajectory. Practically speaking, a large area of scalp provides sufficient engagement, but by deviating significantly from the coronal suture the engagement values tend to diminish.

### Minimum engagement

The lowest recorded engagements for each trajectory are also worth noting; a difference was found when comparing the more anterior trajectories with posterior trajectories (Table 3). Anteriorly, values of 1 or 2 mm were occasionally recorded, whereas posteriorly a minimum of 4 mm was recorded.

### Postoperative stimulation data

We describe eight subsequent patients where the above methodology was used for trajectory planning. Patients underwent mapping of the electrode contacts 6 weeks into the postoperative period to identify side effects before programming (Table 4). Model 3389 (Medtronic, Minneapolis, MN, USA) DBS electrodes were used in all cases. In a 2 × 4 configuration, the deepest contact on the left electrode is labelled as 0, and the deepest contact on the right is labelled 11.

**Table 4:** Side-effect mapping data, in terms of voltage causing capsular side effects, for eight patients whose procedures were planned using the methodology posed by this study

	Patient							
Lead	1	2	3	4	5	6	7	8
0	4.0	4.0	3.0	-	2.5	-	2.0	-
1	4.0	-	3.0	4.0	3.0	-	2.0	-
2	-	4.0	-	4.0	3.5	-	2.0	-
3	-	3.0	-	-	-	-	2.0	-
8	4.0	-	-	-	3.5	-	-	-
9	-	-	-	-	3.5	-	-	-
10	-	-	-	-	-	-	-	-
11	4.0	-	-	-	-	-	-	-

A *hyphen* indicates that no internal capsule stimulation was elicited by the highest voltage tested in that patient (generally 4.0 V but 3.5 V in patient 6 at contacts 1 and 11, and patient 8 at contact 2)

Out of 64 contacts tested, only four contacts in one electrode had a threshold of 2.0 V before the internal capsule was stimulated. In this patient, a lateral trajectory was chosen rather than the central microelectrode tract for electrode placement following intraoperative awake neurophysiology. The lateral tract had a better clinical effect. In retrospect, it seemed that adherence to the central tract would have produced less of a capsular effect and a higher stimulation threshold. Ninety-two percent of contacts had a threshold of 3.0 V or more. All patients could be programmed with therapeutic effects (Table 5).

**Table 5:** Programming settings for eight patients whose procedures were planned using the methodology posed by this study

		Patient							
		1	2	3	4	5	6	7	8
Left	Lead	3	2	2	0	3	1	3	1
	Voltage (V)	2.0	3.0	2.5	2.9	2.4	2.6	1.9	1.0
	Pulse Width (ms)	60	60	60	60	60	60	60	60
	Frequency (Hz)	135	135	135	135	135	130	135	130
Right	Lead	10	10	9	8	10	9	10	9
	Voltage (V)	2.7	3.0	2.5	2.8	2.1	2.6	1.9	1.0
	Pulse Width (ms)	60	60	60	60	60	60	60	60
	Frequency (Hz)	135	135	135	135	135	130	135	130

Programming and mapping data from the first eight patients to be operated upon by our team following the conclusions of this study are provided as early outcome measures.

## Discussion

Our study has confirmed that once a target within the STN is decided, trajectory selection does impact on engagement, as well as margin for error which can arise from either electrode microwobble or brain shift. It would appear that, compared with margin for error, engagement is more uniformly sufficient across our subset of trajectories. Therefore, margin for error would better dictate trajectory selection over more minor variations in engagement within this subset.

As well as accommodating for electrode microwobble, our methodology may be useful when attempting to compensate for brain shift. Brain shift is a possibility in all burr hole-based procedures, but the gross direction and degree of shift are unpredictable. One study reported a 0.6-mm average shift in basal ganglia structures, with 9% of patients exceeding a 2.0-mm shift [2]. Efforts have been made to reduce brain shift such as modifying burr hole techniques [1, 4], but it is considered that trajectory selection could complement these efforts with a greater margin for error when placing each electrode.

**Fig. 3:**
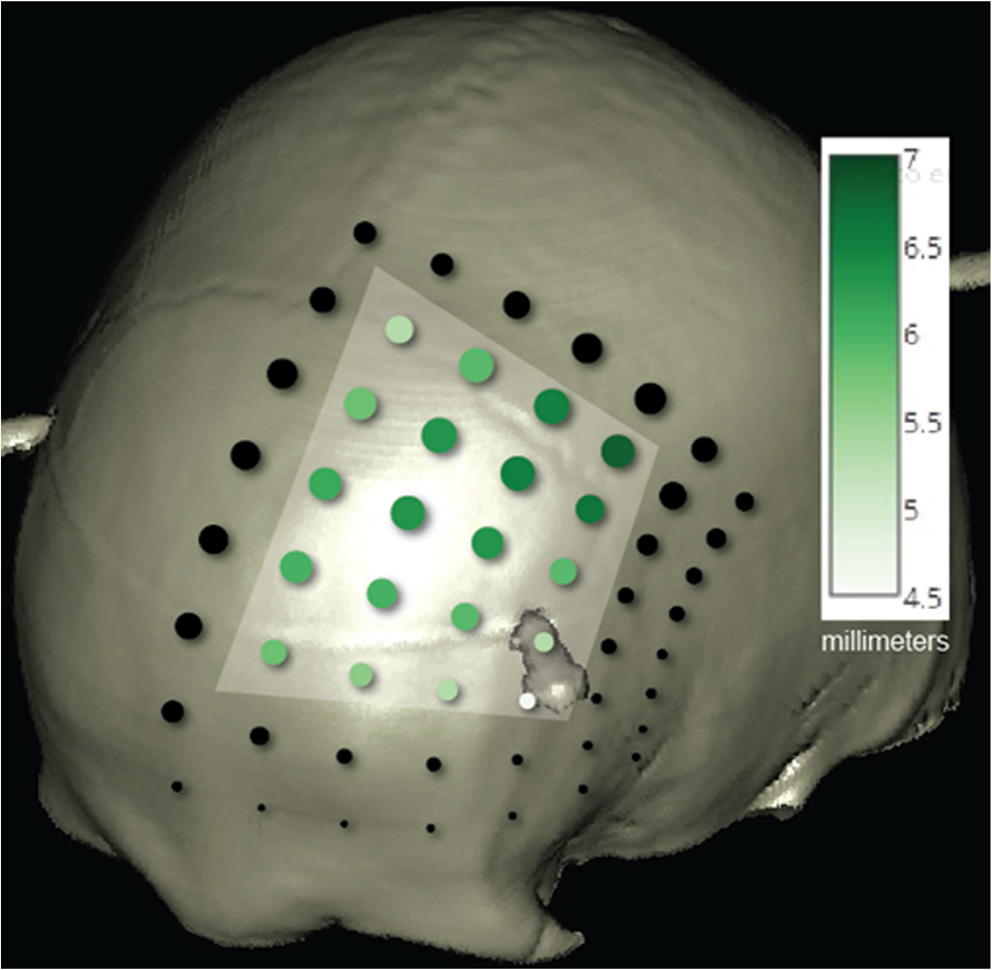
Combined margin for error and length of engagement data, where *each circle* represents one trajectory. The *circle diameter* is proportional to mean margin for error. Within the demarcated subset the *colour gradient* correlates with mean engagement length. (Screenshot from a StealthStation S7 Surgical Navigation System)

The matrix of dots in Fig. 3 represents the 56 trajectories we assessed, combining the data in Tables 2 and 3; the shaded dots are those for which mean engagement was determined, with a darker hue representing longer mean engagement. The diameter of each dot is directly proportional to that trajectory’s margin for error.

We would recommend that surgeons should begin each trial of trajectories around 20 degrees anterior to the bregma and 10 degrees off the midline. Then, in consecutive trials, travel postero-laterally towards the coronal suture and away from the midline—a process which strikes a balance between margin for error and length of engagement. However, where a wide margin for error is paramount, the surgeon may prefer to begin trialling at 20–30 degrees lateral and overlying the coronal suture.

Our methodology has implications for brain shift in bilateral DBS procedures. Despite efforts made to compensate for brain shift by anticipatory targeting, the magnitude and direction of brain shift is unpredictable. This is especially true for the second electrode placement and, therefore, further measures to minimise non-engagement, and thereby clinically suboptimal electrode placement caused by shift, are desirable. When placing the first electrode, the surgeon may choose a tighter trajectory with a small margin for error but considerable engagement. Contralaterally, after some degree of brain shift, they may opt for a trajectory that has a wide margin for error but sacrifices a little engagement—with a view to potentially improving the overall outcome.

Capsular side effect mapping of patients following use of our methodology in planning suggests that the electrode placements were promising; few contacts caused capsular side effects between 2.0 to 3.0 V. Sequential trajectory selection in this manner complements the need to avoid vital anatomical structures by discriminating between unobstructed electrode paths.

## Conclusions

This study sheds light on how trajectory selection affects margin for error and STN engagement. It would appear that the best points of entry are up to 30 degrees anterior to the coronal suture then between 10 and 40 degrees from the midline once a target is fixed. With a view of optimising engagement length we would advise a trial of trajectories that begins at the antero-medial end of this range, passing further trials of trajectories postero-laterally. This should continue until a viable plan is decided that avoids anatomically important structures, whilst achieving an acceptable engagement and margin for error.
